# Effect of Super-Absorbent Polymer (SAP) Incorporation Method on Mechanical and Shrinkage Properties of Internally Cured Concrete

**DOI:** 10.3390/ma15217854

**Published:** 2022-11-07

**Authors:** Xingliang Huang, Xiaoyu Liu, Hongliu Rong, Xiaolong Yang, Yunsheng Duan, Tingting Ren

**Affiliations:** 1 Shaanxi Architecture Science Research Institute Co., Ltd., No. 272 West Ring Road, Xi’an 710082, China; 2 School of Civil Engineering and Architecture, Guangxi University, No. 100, University East Road, Nanning 530004, China

**Keywords:** SAP internally cured concrete, incorporation method, mechanical properties, shrinkage performance, microstructure

## Abstract

To study the effect of SAP incorporation on the early shrinkage of SAP internally cured concrete with the aim to solve the problems of early shrinkage and cracking of bridge leveling-layer concrete, using the SAP incorporation method as a parameter, the mechanical properties test of internally cured concrete, the shrinkage performance test of ring restraint and the scanning electron microscope observation test were carried out. The effects of the SAP content and incorporation method on the flowability, mechanical properties, shrinkage performance and microstructure of internally cured concrete were analyzed. The experimental results show that when the content of the SAP in concrete is 0.2% of the mass of cementitious materials, it has the least influence on the compressive strength of concrete. The addition of preabsorbed water to the SAP can delay early cement hydration, increase the later cement hydration rate and final hydration degree, and improve the concrete strength. Preabsorbed water mixed with an SAP can effectively improve the shrinkage of concrete, and the shrinkage reduction effect is more obvious than that from the dry addition of the SAP; the early cracking risk of concrete without an SAP is high, and it will crack before day 28. The addition of an SAP can strengthen the microstructure of concrete and improve its density and crack resistance, effectively avoiding concrete cracking. It is recommended that the water-absorbent resin be incorporated in a preabsorbent manner, and the SAP preabsorbent ratio is equal to the maximum water storage rate of the SAP.

## 1. Introduction

Cracking caused by concrete shrinkage is an important problem affecting the durability of concrete [1,2,3]. Modern high-strength concrete is characterized by a low water-binder ratio and large shrinkage. The traditional watering curing method generally makes the water stay on the surface of the structure and evaporate rapidly, which does not achieve the purpose of curing. The cement cannot be fully hydrated, and the concrete shrinks greatly, resulting in a large number of cracks [4].

Concrete cracking is affected by many parameters, including the water-cement ratio, cement type, admixture, structural geometry and external conditions [5,6,7]. On the one hand, during the hardening process of cement, due to an insufficient water supply during hydration [8], the internal relative humidity of the cement slurry is reduced, resulting in voids and resulting in increased tensile stress in pore water [9,10]. On the other hand, due to the loss of water in the surrounding atmosphere, the internal relative humidity in the concrete pores decreases, and water loss from the concrete pores will cause stress in the concrete skeleton, resulting in shrinkage [11,12].

The concept of internal curing was first proposed by Bentz [13] in 1991, which incorporates water-absorbing materials such as super-absorbing resins (SAPs) and lightweight aggregates to form a micro-“reservoir” [14] inside the concrete. When the hydration heat of the cementitious material is released, the water on the surface of the concrete evaporates and the internal pores produce negative pressure, and then the “reservoir” will release the water stored in the water-absorbing material to promote the full hydration of the cement and achieve the curing effect [15,16,17].

The shrinkage of concrete is obviously reduced after adding a water-absorbing material [18,19,20], and especially after adding an SAP with a high water absorption rate, the concrete will expand slightly in the early stage [21,22]. An SAP incorporated into concrete first absorbs water to expand and then releases water to shrink. The SAP releases moisture to effectively reduce concrete shrinkage, but also forms defective holes in the concrete, reducing the strength of the concrete [17,23,24].

At present, there is much research on the shrinkage cracking of concrete. Most scholars have studied the effect of SAP incorporation on mechanical property and concrete shrinkage performance [1,19]. Yu et al. [25] proved that the shrinkage and tensile strength of UHPC increased first and then decreased with the increase in the SAP content by using a three-point bending test of a notched beam, a single-crack tensile test and mercury porosimetry; they found the optimum content of SAP to be 0.1~0.15%. Laurence et al. [26] conducted a bellows test and a restrained ring test to prove that an SAP can reduce the autogenous shrinkage of a mortar mixture by more than 80% within 7 d, and the shrinkage reduction effect is weakened after 7 d. Yang et al. [27] studied the effect of an SAP on the drying shrinkage and humidity distribution of concrete slabs, proving that the SAP had the best effect of inhibiting shrinkage within 7 d, and the internal curing of the SAP greatly reduced the humidity difference in pavement slabs at different depths. Yuka et al. [28] developed a delayed-absorption SAP, which can reduce autogenous shrinkage without adding additional water and does not affect compressive strength.

In view of the influence of different incorporation methods of the SAP, the main direction of existing research is focus on the SAP’s influence on the rheological properties of concrete. Liu et al. [29] evaluated the influence of two kinds of SAPs on the rheology of UHPC mortar under different particle sizes and dosages, and found that the pretreatment method of the SAP had no significant effect on thixotropy; Ma et al. [30] examined the effects of a dry SAP on the maximum shear stress and yield stress of cement-based materials, and found that the dry SAP increased yield stress and plastic viscosity; Adams et al. [31] developed a simple mixing design method for the formulation of 0.42 w/c ordinary Portland cement mortars, requiring the addition of additional water or a high range of water-reducing agents when the SAP is 0.2% of the cement weight.

In summary, most of the studies on the influence of an SAP on the concrete shrinkage performance at present focus on the influence of the SAP type and content on concrete shrinkage cracking. In view of the influence of different SAP incorporation methods on concrete properties, most of the research focuses on how to determine the additional water content of the SAP to improve the rheological properties of cement-based materials. It can be seen that, at present, under different SAP incorporation methods, and especially under the condition of SAP partial pre-water absorption, the influence on concrete mechanics and shrinkage properties is still small.

Based on the above reasons, this paper takes the SAP incorporation method as the parameter and considers the change law of water absorption over time in the concrete mixing stage to carry out the flow performance test, compressive strength test and constrained shrinkage performance test of internally cured concrete. The effects of different SAP incorporation methods on the fluidity, mechanical properties and ring shrinkage strain of internally cured concrete were studied. The degree of hydration of concrete under different SAP incorporation methods was analyzed by using scanning electron microscopy experiments, and the internal hydration mechanism of concrete under different SAP incorporation methods was explained in order to provide a reference for the application of SAP internally cured concrete.

## 2. Raw Materials and Test Methods

### 2.1. Raw Materials

#### 2.1.1. Cement

The cement used was P • O 32.5 ordinary Portland cement, according to the “general Portland cement” GB 175-2007 [32]. The cement requirements to test for the indicators were met by the basic physical properties of cement, which are shown in Table 1.

#### 2.1.2. Aggregate

The particle size of the coarse aggregate used in the test was 4.75~19.5 mm, and the mixing ratio of 4.75~9.5 mm and 9.5~19.5 mm was 4:6. The crushing value of the crushed stone was 17.5%, the needle flake content was 4.3% and the mud content was 0.12%.

The fine aggregate used in the test was machine-made sand, and the fineness modulus was 2.5. According to the interval of the cumulative sieve residue curve, this kind of fine aggregate is a medium sand with good gradation.

#### 2.1.3. Internal Curing Agent

The internal curing agent used in the test was a super-absorbent resin (SAP), which is a highly crosslinked sodium acrylate produced by aqueous solution polymerization (crosslinking agent content of 0.06%), and the average particle size was 150 μm.

#### 2.1.4. Water and Water Reducer

The water used in the test was tap water. 

The admixture used in the test was a polycarboxylate powder concrete superplasticizer, the water reduction rate was 30% and the bleeding rate ratio was ≤20%.

### 2.2. Mix Design

Because of the inability to accurately measure the amount of water released by the SAP inside concrete, researchers have measured the water absorption kinetics curve of the SAP in cement-based centrifugal fluids. The results show that the development law of SAP water absorption in a cement slurry centrifugal fluid is divided into three stages: first, it rises rapidly to the maximum, then gradually decreases, and finally, tends to be stable [33,34]. Studies have found that before the final setting of concrete, the concentration of the pore solution of cement paste does not fluctuate much, generally staying around 0.7 mol/L, and a 0.7 mol/L sodium chloride solution can be used to simulate a cement-based solution [35].

Firstly, the presaturated water-absorbing SAP was placed in a 0.7 mol/L sodium chloride solution to simulate the change process of the water absorption rate of the SAP in a concrete mixture. The tea-bag method [34,36,37] was used to measure the maximum water absorption rate of the SAP per unit mass in the process of pre-water absorption (u_1_ = 146.02) and the water storage rate of the preabsorbed SAP when the concentration of the pre-water absorption SAP in the sodium chloride solution was balanced (u_3_ = 35.00). The u_2_ (u_2_ = 111.02) is the water release rate of the SAP in the concrete mixing stage. Considering the change in the water absorption rate of the SAP in the concrete mixing stage, the SAP pre-absorption water and concrete unit water consumption rates of each test group were calculated. The mix proportion design results of internally cured concrete are shown in Table 2 (W_0_ is the pre-water absorption of the SAP, and the content of the SAP is 0.2% of the mass of cementitious materials). Six kinds of mix proportions were designed: the J0 group without an SAP, the G0 group with a dry SAP, the G1 group with a dry SAP and additional m × u_3_ water, the Y0 group with a pre-saturated SAP and reduced water consumption of m × u_2_, the Y1 group with a pre-saturated SAP and reduced water consumption of m × u_2_, and the Y2 group with m × u_3_ pre-water absorption.

### 2.3. Experimental Design

#### 2.3.1. Flow Test of Concrete

The maximum particle size of the aggregate used in the preparation of the internally cured concrete was 19 mm. The visual slump of concrete was much larger than 10 mm during the trial mixing. Therefore, according to the slump measurement method in the “Ordinary concrete mixture performance test method standard” GB/T 50080-2002 [38], the flow performance of the internally cured concrete mix with three kinds of SAP contents (0.1%, 0.2%, 0.3%) was measured.

#### 2.3.2. Mechanical Performance Test of Concrete

According to “the test method of mechanical properties of ordinary concrete” GB/T 50081-2002 [39], three sets of standard cube specimens with a size of 150 mm × 150 mm × 150 mm were made, with three in each group. The compressive strength of concrete with three kinds of SAP contents (0.1%, 0.2%, 0.3%) was measured to determine the optimal content of the SAP. The compressive strength of the concrete at key ages (1, 3, 7, 14, 28, 60, 90 d) was measured under different SAP incorporation methods, and the development law of the compressive strength of internally cured concrete with concrete curing age was studied.

#### 2.3.3. Experiment on Restrained Shrinkage Performance of Concrete Ring

According to ASTMC 1581-04 [40], the annular restraint shrinkage test of concrete was carried out. The height of the ring was 100 mm, the outer diameter of the inner steel ring was 305 mm and the thickness was 5 mm. The thickness of the concrete ring to be poured was 60 mm, and the bottom circular steel plate had a groove to limit the inner and outer steel rings. The half-bridge patch method was used to evenly paste strain gauges along the inner surface of the inner steel ring in the annular direction. The strain gauges were the A120 type with built-in temperature regulation, and there were four strains in total. The strain gauges were connected to the static strain collection instrument. The timing began with the completion time of the casting. The stress change was recorded every 6 h on the first d, and the stress change and crack development in the concrete ring were recorded every 12 h thereafter until the end of 28 d.

#### 2.3.4. Microscopic Test of Scanning Electron Microscopy

After the shrinkage test, the concrete specimen was taken out and broken. The cement matrix fragments were screened and soaked in an alcohol solution to terminate the hydration process of the internal cement. After drying with a dryer, the processed dry test block was pasted on conductive glue. After the gold sputtering, the samples were put under the scanning electron microscope (The instrument is a FEI Quattro S model from the United States Thermoelectric Company, produced in the Czech Republic) to observe the morphology of cement hydration products and cracks.

## 3. Results and Discussion

### 3.1. Concrete Fluidity Test Results

**Figure 1:**
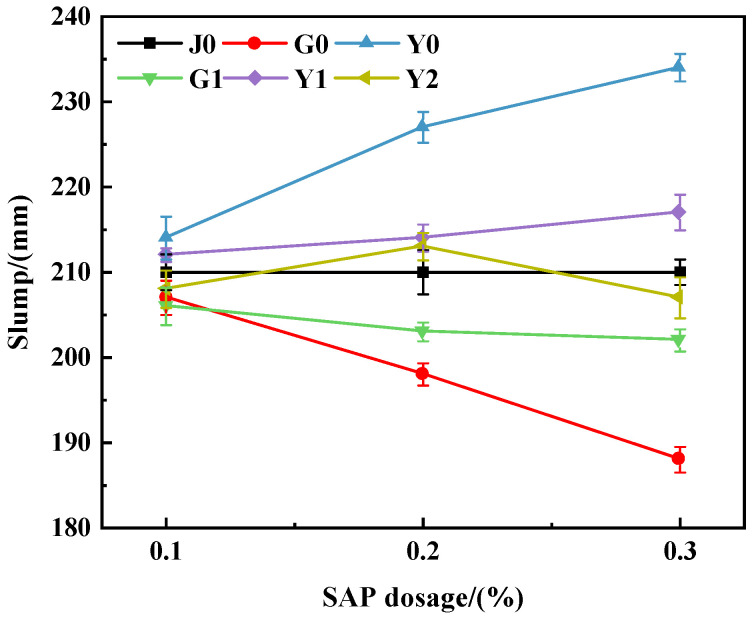
Concrete slump with different SAP contents.

Due to the high water absorption rate of the SAP, the working performance of the concrete will be greatly affected when it is mixed into concrete with dry or pre-water absorption. Therefore, the flow performance of SAP internally cured concrete is studied. The measured concrete slump under different SAP contents and incorporation methods is shown in Figure 1.

As can be seen from Figure 1, under the five incorporation methods, the slump of the Y0 group was significantly higher than that of the J0 group and showed an obvious increasing trend with the increase in SAP content; the slump of group G0 was significantly lower than that of group J0, and decreased with the increase in SAP content; the slump was slightly reduced in the G1 group; and the slumps of the G1, Y1 and Y2 groups were similar to that of the J0 group. 

Compared with the reference group (J0), the slump of the dry-mixed group (G0) and the dry-mixed water group (G1) decreased with the increase in the amount of absorbent resin, and the decreases were 1.5%, 6% and 10.5% and 1.9%, 3.4% and 3.8%, respectively. When a dry SAP is added to the mixture, the SAP absorbs water from the slurry, reducing the effective water content and w/b, which is equivalent to reducing water [41]. Without providing additional water, it will lead to an increase in yield stress and plastic viscosity, and lead to a decrease in slump flow and an increase in mortar flow time [42,43,44]. When the SAP is used in a dry state, additional water should be added to make up for the water absorbed by the SAP during the mixing process so that SAP does not have an adverse effect [42]. Compared with the G0 group, the G1 group added extra water to make up for the water absorbed by the SAP from the cement slurry, and thus, the slump decreased less and the flow performance was better. Due to the complex ion composition of the concrete cement slurry, the SAP cannot absorb all the additional water, and cement and fine aggregate particles are adhered around SAP particles, which increases the friction between aggregates; therefore, the slump of the G1 group is lower than that of the J0 group.

For the Y0 group with presaturated water absorption and additional water, the slump increases by 1.9%, 8.1% and 11%. The SAP release of water in the Y0 group increases the water-cement ratio and makes the concrete fluidity increase significantly. Compared with the reference group (J0), the slump of the water-saturated and water-reduced group (Y1) was slightly increased; the reason was that the preabsorbed absorbent resin absorbed less or no longer absorbed the mixed water and cement slurry, and the absorbent resin was evenly dispersed in the concrete, meaning that a small amount of water would be released, and thus, the fluidity of the concrete did not change much. The partial preabsorbent SAP (Y2) did not absorb water or release water after mixing with the mixture; thus, compared with group J0, the slump of the concrete also remained basically unchanged. Some studies [29] have confirmed that the addition of a preabsorbed SAP has no significant effect on the flow time of the mini-V-shaped funnel of UHPC, indicating that a preabsorbed SAP has little effect on the fluidity of different kinds of concrete mortars.

### 3.2. Concrete Compressive Strength Test Results

**Figure 2:**
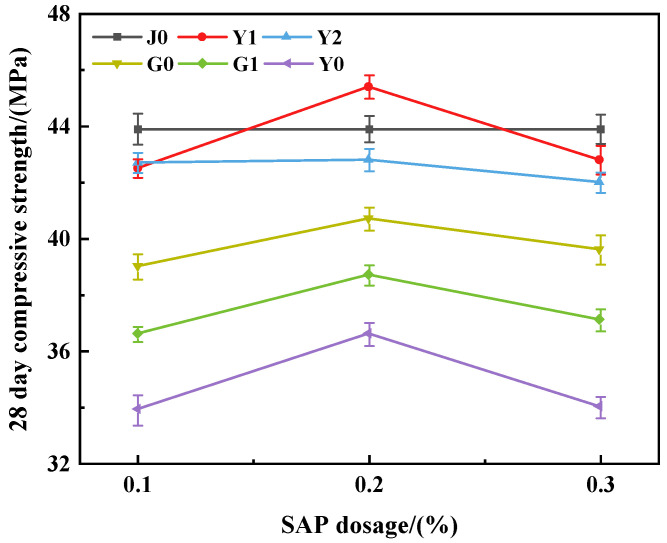
The 28 d compressive strength of concrete.

The compressive strength of concrete with different SAP dosages and incorporation methods is shown in Table 3 and Figure 2.

According to the analysis of the data in Table 3, the average value of the 28 d compressive strength of concrete with 0.2% SAP content is the highest, and the fluctuation is relatively small; the relative decrease amplitude of compressive strength in the Y1 and Y2 groups was the smallest, and the pre-water absorption in the Y2 group was weakly sensitive to the SAP content.

As can be seen from Figure 2, the compressive strength curves of group Y1, group G0, group G1 and group Y0 all showed regularity. With the increase in the SAP content from 0.1% to 0.3%, the compressive strength first increased and then decreased, and the compressive strength reached the peak when the SAP content was 0.2%. It can be considered that an SAP of this type has the least influence on concrete strength when the content is 0.2%. This may be due to SAP water absorption characteristics leading to a decrease in the effective water-cement ratio, resulting in increased strength, and the strength increase caused by the decrease of water-cement ratio exceeds the strength decrease caused by SAP shrinkage. It can be considered that 0.2% SAP content is the best dosage, which is similar to the finding in the research of Il-Sun Kim [45], and the best dosage of different types of SAPs will be different.

After the SAP content exceeds 0.2%, the strength of each group generally decreases. It is speculated that the main reason may be the uneven distribution of water in the matrix caused by the addition of excessive SAP [46]. Subsequently, due to the large number of pores in the concrete during the hardening stage, the compressive strength decreases [47,48,49]. The compressive strength of G0 and Y2 decreased due to the formation of holes in the concrete by the SAP. The strength of the G1 group and Y0 group decreased, on the one hand, because the incorporation of the SAP formed holes in the concrete; on the other hand, it was also because additional water or the SAP’s additional release of water reduces the water-binder ratio of concrete, thereby reducing the strength of concrete.

In summary, when the SAP content is low, its water storage and release function are not obvious, the internal curing effect is not obvious, and the compressive strength of the concrete increases slightly. When the SAP content is too high, the holes left after the shrinkage of the SAP and the release of water become the main factor affecting the compressive strength of the concrete, which reduces the compressive strength of the concrete. Therefore, under the conditions of appropriate dosage, an appropriate water diversion method and appropriate water diversion, the SAP internal curing effect can be the best, and the strength of the concrete can reach its maximum.

### 3.3. Test Results of Compressive Strength of Concrete at Different Curing Ages

**Figure 3:**
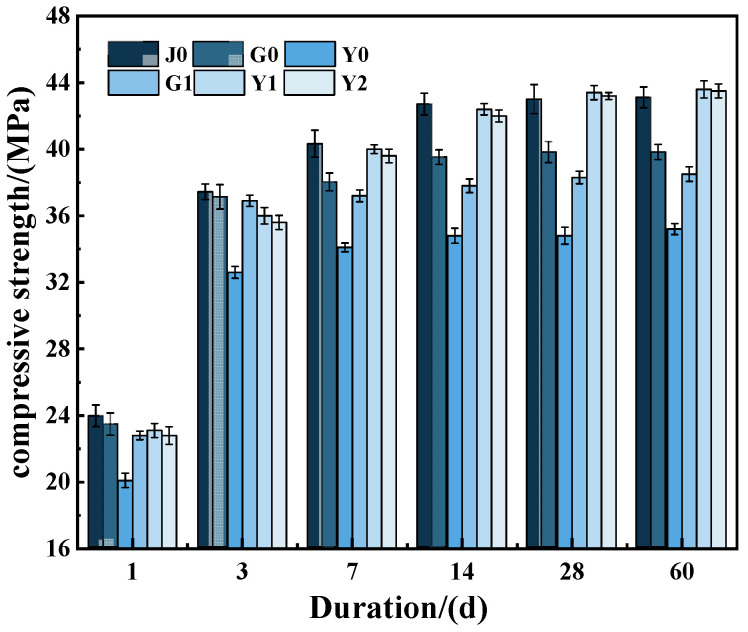
Development of concrete strength under different SAP incorporation methods.

The compressive strength test was carried out with the optimal dosage of 0.2% (the SAP content has the least effect on the strength of concrete, and the SAP content in the follow-up test is 0.2%). The variation law of compressive strength of internally cured concrete with curing ages under different SAP incorporation methods is studied. The results are shown in Figure 3.

As can be seen from Figure 3, with the increase in the curing age, the strength of the concrete develops rapidly in the first 7 d, but slows down after 7 d. The compressive strength of concrete at 28 d reaches more than 95% of that at 90 d, and the growth range of the compressive strength is very small.

The development trend of the compressive strength of group Y1 and group Y2 is relatively consistent. In the early stage, the intensity development was slower, whereas in the later stage, the intensity development speed was faster, but the overall difference was small. The development trend of the compressive strength of the G0 group and G1 group was basically the same, which shows that the early strength developed faster and the late strength developed slower; the 7-d compressive strength reached 90% of the 28-d compressive strength, and the later compressive strength growth was only 2.7 MPa and 2.6 MPa, respectively, which finally stabilized at 39.8 MPa and 35.2 MPa. The compressive strength of the Y0 group was consistently lower than that of the other five groups; the 7-d compressive strength reached 80% of the 28-d compressive strength, and the later compressive strength increased slowly with age.

The intensity development trend of the G0 group and G1 group was similar. The main reason is that these two groups of SAPs have the same mechanism of action. After adding water, the water-binder ratio of the G1 group increased and the maximum strength decreased in the later stage. However, the incorporation of the SAP is consistent, and thus, the change trend of strength with age was consistent. Secondly, the dry, mixed SAP absorbed the cement slurry, which is equivalent to reducing the water-binder ratio of the concrete. The hydration heat of the cement was intensified, and the water in the capillary pores of the concrete dissipated faster; therefore, the early strength is high and the later strength is small. Finally, in the cement hardening stage, the SAP water-release shrinkage left holes in the concrete, and the concrete compressive strength decreased, making its 90-d compressive strength lower than that of the J0 group.

The compressive strength of the Y0 group was lower. The reason may be due to the fact that in the concrete mixing stage, the ion concentration of the cement slurry is greater than the ion concentration inside the SAP particles of the Y0 group. Therefore, the compressive strength is low in the early hardening stage. At the same time, it also leads to an uneven distribution of water in the concrete, resulting in large pores [46]. Hydration products generated by hydration are not enough to make up for the strength loss caused by pore expansion. 

The strength development trend of group Y1 and group Y2 was similar. In both groups, the SAP was preabsorbed so that sufficient water was stored inside the SAP crosslinked network structure. The water absorbed by the saturated SAP in the Y1 group is quickly released during the concrete mixing process. With the increase in the water-cement ratio, the early compressive strength decreased. After the initial cement setting, the gradual release of the additional water stored in the SAP helps to form a denser and more homogeneous cementing matrix, which acts as an effective filler for the voids near the aggregate and ensures the dense filling of cement particles [50], thus resulting in a slightly greater increase in compressive strength at a later stage than in the J0 group. Therefore, the growth range of the compressive strength in the later stage is slightly greater than that of the J0 group. 

From the overall test results of compressive strength, it can be concluded that pre-water absorption of the water-absorbing resin has a better effect on the inner curing of concrete than dry incorporation. SAP presaturated water absorption and water consumption reduction have the same internal curing effect as SAP partial pre-water absorption.

### 3.4. Shrinkage Performance of Concrete

**Figure 4:**
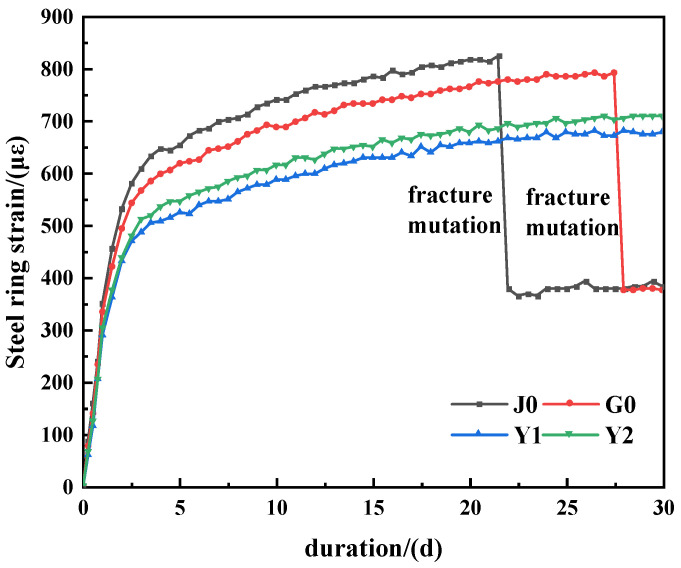
The strain value of the steel ring.

With 0.2% SAP content, the shrinkage strain of the concrete ring under different SAP incorporation methods was studied. The steel-ring strain test results of different concretes are shown in Figure 4, and the concrete cracks are shown in Figure 4. 

It can be seen from Figure 4 that with the increase in curing age, the circumferential compressive stress of the inner steel ring increased gradually, and the initial microcracks were observed in the J0 group and G0 group. It can be seen from the strain curves of the steel ring that the stress of the steel ring decreased after adding the absorbent resin, which indicates that adding an absorbent resin can effectively reduce the shrinkage of concrete; the reduction was 25% in group G0 and 30% in groups Y1 and Y2. 

**Figure 5:**
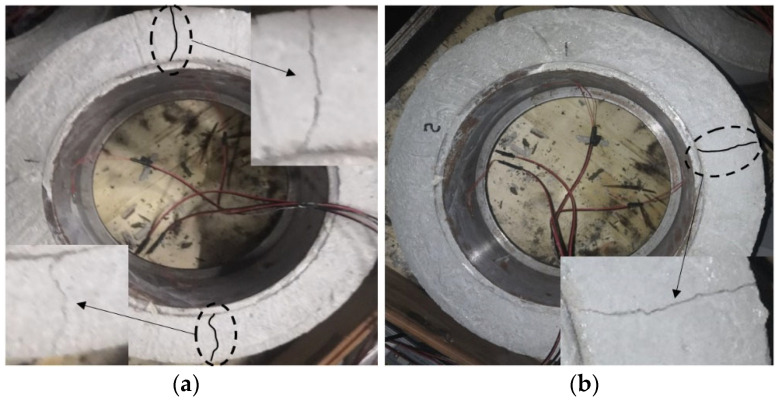
Cracks in concrete rings (the left is J0, and the right is G0). (**a**) Two cracks appeared in the concrete ring; (**b**) A crack appeared in the concrete ring.

According to reference [26], the development of the shrinkage of cement materials can be divided into three stages: the liquid stage, skeleton formation stage and final hardening stage. The volume change caused by the early contraction is more significant. Judging from the development of the concrete shrinkage strain, the first 7 d is the rapid shrinkage stage of concrete, in which the shrinkage stress of concrete develops rapidly. After 7d, the initial setting of concrete is over, and the stress enters the stable development period. After 22 d, the ductility of the J0 concrete reached the limit and the stress of the steel ring changed abruptly, decreasing from 825 microstrain to 379 microstrain. The concrete ring changed from an initial microcrack to final crack, and two penetration cracks appeared (as shown in Figure 5). At about 28 d, the concrete ring of group G0 cracked and a penetrating crack appeared. The stress of the steel ring changed abruptly, decreasing from 793 microstrain to 376 microstrain, and the concrete ring crack appeared. However, the steel-ring stress of the Y1 and Y2 groups increased rapidly before 7 d and remained stable after 7 d, and no cracks were observed in the concrete ring.

The reason for the above phenomenon is speculated to be that the steel-ring stress gradually increases with the concrete contraction, and the strain values of the three experimental groups are all lower than those of the J0 group, indicating that the incorporation of the SAP effectively reduces the concrete contraction. Studies have shown that the incorporation of an SAP will change the texture of concrete [51] and improve its workability, consistency and plasticity [52]. A gel material provides cushioning, which helps to improve the stability of fresh concrete [53], and the increase in the polymer volume helps to seal cracks inside concrete [54]. Incorporation of a preabsorbed SAP can effectively store water in the early stage, and provide sufficient water for internal curing in the later stage of concrete curing, which slows down the hydration process inside the concrete. The splitting tensile strength of concrete develops rapidly, whereas the shrinkage stress in the concrete ring develops slowly. Therefore, compared with the J0 group, the stress of the steel ring in groups Y1 and Y2 decreased greatly and developed slowly, and the concrete ring did not crack until d 30. The dry SAP absorbs the cement slurry at the early stage of concrete hardening and reduces the fluidity of the concrete mixture, which is equivalent to reducing the water-binder ratio of the concrete, making the SAP release water too early and too fast in the later period of maintenance, reducing the internal curing effect of the SAP. That is, the steel-ring stress of the G0 group is slightly lower than that of the J0 group.

### 3.5. Microscopic Test Analysis Using a Scanning Electron Microscope

**Figure 6:**
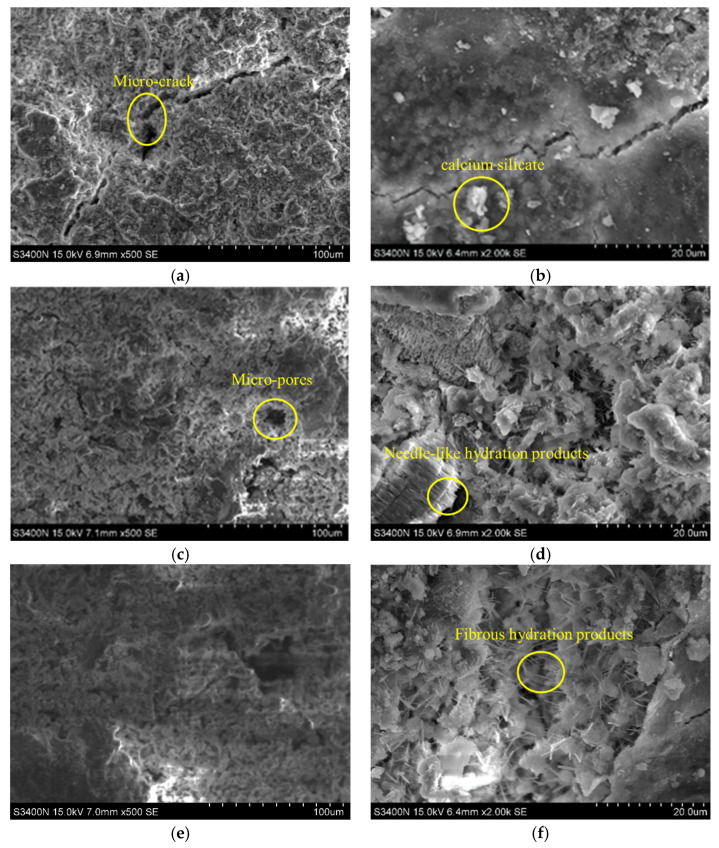
SEM image of concrete sample. (**a**) J0 (×500); (**b**) J0 (×2000); (**c**) G0 (×500); (**d**) G0 (×2000); (**e**) Y1 (×500); (**f**) Y1 (×2000).

A scanning electron microscope (SEM) was used to observe the hydration products of cement and the changes in the microstructure of the cement matrix after incorporation of the SAP. The reasons for the change in mechanical properties and the crack resistance of internally cured concrete were analyzed. The results are shown in Figure 6.

The microstructure of hardened cement is mainly composed of the following phases: a calcium silicate hydrate phase, calcium hydroxide, an unhydrated binder and an aggregate [55,56]. It can be seen from Figure 6 that there is a small amount of calcium silicate hydrate gel and a small amount of rod-like ettringite on the cement matrix of group J0, and there are obvious microcracks in the cement matrix. There are a large number of micropores on the surface of the matrix, and the diameter of the pores is relatively large, the structure is relatively loose and the density is relatively poor. There are still a large number of pores on the surface of the matrix, in group G0 with dried SAP, but the number of pores is less and the size of the pores is smaller than that of group J0. The hydration products on the matrix surface are mostly needle sheets. In group Y1, there is still calcium silicate gel on the cement matrix, but compared with the blank control group, the number and size of holes were further reduced. In addition, a large amount of stout, fibrous ettringite grows on the surface walls and cracks of the matrix, and the pore area ratio of the matrix decreases, and thus, the cracks also decrease significantly.

SAP cement-based materials have been proven to have self-healing properties [57,58,59]. When an SAP is incorporated into cement-based materials, the SAP gradually releases water, which reacts with the surrounding unhydrated cement, resulting in hydration products that can fill and heal microcracks. In addition, the entry of water causes the SAP to expand, and then the expanded SAP gel fills the macroscopic cracks and restricts water flow. According to some studies, when water flows through cracks, SAP particles near the downstream surface become dry, whereas particles near the upstream surface expand to form soft gels and fill SAP voids and cracks [58]. The longer the cement hydration process, the more hydration products are generated, and the more robust the morphology will be. The size of hydration products in group Y1 is larger than that in group J0, indicating that the reservoir function of the preabsorbed SAP plays a better role, prolonging the hydration time and making the hydration reaction of cement more adequate and the matrix structure more compact.

Therefore, the incorporation of an SAP slows down the early hydration rate, and the cement is further hydrated after water release; thus, water is released in the later stage of cement hardening and the hydration process is prolonged, which improves the compacting degree of concrete and enhances its crack resistance.

**Figure 7:**
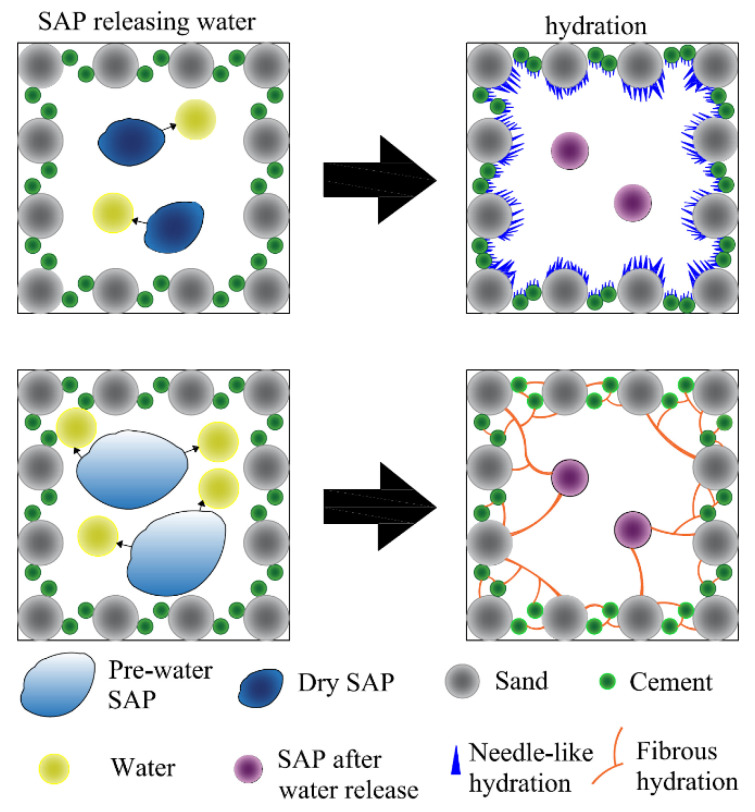
Hydration mechanism of SAP internally cured concrete.

Figure 7 shows the internal hydration reaction mechanism of concrete with different SAP incorporation methods.

As can be seen from Figure 7, the different effects of the SAP incorporation methods mainly lie in the strength loss caused by the SAP releasing water into pores and the strength increase caused by the hydration degree. The SAP supplies preabsorbed water to the pores when they dry, thereby reducing the capillary force [41]. Water is usually absorbed by the SAP and released after the concrete solidifies [60]. Therefore, the SAP promotes the hydration of cement [61] while shrinking and leaving gaps of more than 1000 nm in cement-based materials [62] and weakening the effective cross-sectional area of the compressed surface, which has a negative impact on the compressive strength [63]. These two effects have opposite effects on the mechanical properties of concrete. Therefore, the effect of SAP incorporation on compressive strength depends on the strength of both influences.

When the SAP is dried and incorporated, the cement slurry will be absorbed in the mixing stage, resulting in the reduction in the water-cement ratio of the mixture. However, after the initial setting of concrete, the internal water content of concrete is low, and the SAP releases water prematurely; thus, the hydration process is shorter, and the early strength of the concrete is high and the later strength is low. At the same time, premature SAP water release also weakened the improvement in the concrete shrinkage. When the SAP is preabsorbed and incorporated, the water-binder ratio is reduced during the mixing phase, resulting in an increase in the water-cement ratio of the mixture. In the late curing period, the SAP releases enough water to delay the hydration rate so that the cement is fully hydrated and enough hydration products are generated to make up for the pores produced by SAP shrinkage. Therefore, the early strength of the concrete is low and the late strength is high, and the concrete crack resistance performance is greatly improved.

## 4. Conclusions

(1) The SAP incorporation method has a significant effect on concrete fluidity. The fluidity of concrete increases when a preabsorbent SAP is added, and is positively correlated with SAP water content and SAP content. The fluidity of concrete decreases when the SAP is dried and is negatively correlated with SAP content.
(2) It can be considered that the optimum content of this type of SAP in concrete is 0.2%. The compressive strength of concrete mixed with a preabsorbent SAP has a relatively low development before 28 d, and a relatively large increase after 28 d.
(3) The shrinkage property of concrete mixed with an SAP is improved and the shrinkage strain is significantly reduced. The shrinkage strain of concrete mixed with a pre-absorbent SAP decreases the most, and no cracking occurs until the end of the test. The shrinkage-reducing effect of a dry SAP is limited and concrete still cracks.
(4) The SAP stores water in the early stage of hydration, and releases water in the later stage to promote hydration. Therefore, the compactness of concrete is improved and the crack resistance is improved. The shrinkage reduction effect of a preabsorbent SAP is better than that of a dry SAP. The pre-water absorption method is recommended, and the pre-water absorption ratio is the maximum water storage rate of the SAP.

## Figures and Tables

**Table 1:** Physical properties of P • O32.5 ordinary Portland cement.

Apparent Specific Gravity	Specific Surface Area (m^2^/kg)	Stability	Setting Time (min)		Strength (MPa)			
					Flexural		Compressive	
			Initial	Final	3 d	28 d	3 d	28 d
3.1	330	Qualified	201	274	2.8	5.7	18.5	37.2

**Table 2:** Internally cured concrete mix ratio.

	SAP /(g/m³)	W_0_ /(kg/cm³)	W /(kg/m³)	Cement /(kg/m³)	S /(kg/m³)	G /(kg/m³)	Water-Reducing Agent
J0	0	0	165	470	668	1100	0.04%
G0	904	0	165	470	668	1100	0.04%
G1	904	0	196.7	470	668	1100	0.04%
Y0	904	132.01	165	470	668	1100	0.04%
Y1	904	132.01	89.70	470	668	1100	0.04%
Y2	904	31.74	165	470	668	1100	0.04%

**Table 3:** Mean and variance analysis of compressive strength.

Group	Average	Variance
0.1%	39.77	14.64
0.2%	41.35	11.00
0.3%	39.90	14.31
J0	43.90	0.00
Y1	43.57	2.54
Y2	42.50	0.19
G0	39.77	0.74
G1	37.47	1.20
Y0	34.83	2.34

## Data Availability

The data presented in this study are available on request from the corresponding author.
